# Functional Assessment Over Time Using Electroretinograms: A Case Report of a Pediatric Patient With Central Retinal Artery Occlusion

**DOI:** 10.7759/cureus.90696

**Published:** 2025-08-21

**Authors:** Yuro Igawa, Kazuhiro Ueyama, Hiroshi Ishikawa, Junji Kanno, Midori Tachibana, Kei Shinoda

**Affiliations:** 1 Department of Ophthalmology, Saitama Medical University, Saitama, JPN

**Keywords:** central retinal artery occlusion, crao, electroretinography, erg, retina

## Abstract

This case report describes a rare instance of central retinal artery occlusion (CRAO) in a 10-year-old girl, emphasizing the importance of electroretinography (ERG) in monitoring retinal function over time. CRAO, which is typically prevalent in middle-aged and older adults, manifests abruptly with painless vision loss and is seldom reported in the pediatric population, making this case particularly noteworthy. Unlike adults, whose CRAO often stems from atherosclerosis or emboli, pediatric cases may involve different etiologies, such as trauma or congenital defects like atrial septal defects. In this case, the patient experienced sudden vision loss in her right eye following ocular trauma. Initial examinations showed a cherry red spot and retinal swelling, with normal anterior segments and visual acuity of 0.4 in the affected eye. Over a period of six months, assessments included cone ERG, which documented a decline in both a-wave and b-wave amplitudes, indicative of progressive retinal dysfunction affecting the inner layer. Despite the retinal degeneration, the patient's visual acuity improved slightly, likely due to the collateral circulation from the ciliary artery, which is not typically affected by CRAO. This case underscores the utility of ERG in providing a comprehensive evaluation of retinal health that cannot be discerned from visual acuity alone. The findings suggest that despite the structural damage, certain retinal functions can be preserved, offering insights into potential therapeutic targets. This case adds valuable information to the limited literature on pediatric CRAO and supports the need for further research to better understand its pathophysiology and management. The role of the ciliary artery in maintaining visual function despite central retinal artery compromise is a salient area for future studies, with the potential to inform treatment strategies for preserving vision in similar cases.

## Introduction

Central retinal artery occlusion (CRAO) is one of the most severe retinal vascular occlusive diseases, characterized by sudden, painless loss of vision and involving a variety of intrinsic and systemic factors [1,2]. In pediatric patients, sudden visual loss accompanied by visual field defects may be caused by conditions such as optic neuritis, ischemic optic neuropathy, vasculitis, or retinal ischemia secondary to trauma. However, CRAO is exceedingly rare. The central retinal artery enters the eye through the optic nerve head and is the primary vessel supplying oxygen and nutrients to the inner retinal layers. In CRAO, the blood flow to the inner retinal layers is acutely interrupted, leading to a cessation of oxygen supply to the photoreceptors and causing a sudden loss of vision. The electroretinogram (ERG) is a pivotal diagnostic tool for assessing retinal function over time [3]. It is used as an objective tool to assess the function of individual retinal layers, including photoreceptors and bipolar cells [3]. In CRAO, ERG has been utilized to evaluate visual function objectively and to predict visual prognosis [4]. In patients with CRAO, a decrease in b-wave amplitude is indicative of inner retinal ischemia at a single flash ERG of 40 J from a xenon lamp recorded after 20 min of dark adaptation [5]. As CRAO is known to occur most commonly in middle-aged and older adults, its occurrence in children is extremely rare, and there are limited reports in the literature [6].

The pathophysiology of CRAO in children is not fully understood and may differ significantly from that in adults. While adults share many common risk factors, such as atherosclerosis and embolism, trauma, carotid artery dissection, and atrial septal defect have been identified as potential factors associated with CRAO in pediatric cases [7-10]. Because these differences influence diagnostic and therapeutic approaches, case reports of pediatric CRAO are important for a better understanding of this disease.

In this report, we describe a case of CRAO in a 10-year-old girl in which temporal changes in retinal function were recorded over a six-month period using cone ERGs, which adhere to the International Society for Clinical Electrophysiology of Vision (ISCEV) standard [11]. As a rare pediatric case, this case is intended to contribute to the discussion of clinical management and prognosis and to demonstrate that the evaluation of retinal function using ERGs is useful in understanding the mechanisms of disease progression and dysfunction in pediatric CRAO.

## Case presentation

The patient, a 10-year-old girl, presented to our department with a chief complaint of sudden loss of vision in her right eye. Her symptoms began after she returned home from elementary school, when her right eye started itching and she rubbed it. Since then, she has noticed a decline in her vision in her right eye. Her past medical history included childhood asthma and spider angioma, and there was no notable family history. The patient reported allergies to house dust, cats, and dogs and was taking oral olopatadine hydrochloride.

**Figure 1:**
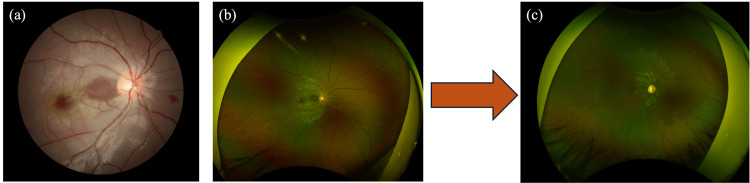
Fundus imaging at the first visit. At the first visit, the right fundus showed a cherry red spot and diffuse retinal pallor with residual hairy retinal arteries; six months later, the cherry red spot and diffuse retinal pallor had improved, and the optic nerve papillae had become more pallid.

At the time of the initial examination, the visual acuity was 0.4 in the right eye and 1.2 in the left eye. The intraocular pressure was 14.0 mmHg in the right eye and 15.7 mmHg in the left eye (Figure 1). 

**Figure 2:**
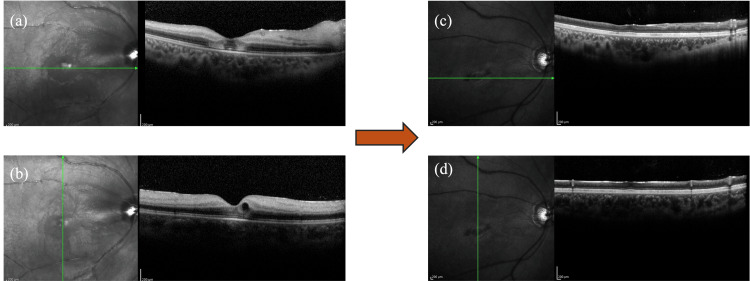
OCT Optical coherence tomography at the first visit. OCT: Optical coherence tomography At the first visit, the inner retinal layer edema of the right eye was noted; six months later, the retina was atrophic and thinning.

Fundus photography also clearly demonstrated a patent cilioretinal artery running from the optic disc toward the macula, which is considered to contribute to the preservation of central vision in such cases. No obvious abnormalities were observed in light reflection or relative afferent pupillary defect (RAPD). The ophthalmological examination revealed no abnormalities in the anterior segment or the intermediate media, but while the entire retina appeared whitish due to edema, the coloration of the fovea was preserved, and a cherry red spot was observed in the fundus of the right eye. No abnormalities were noted in the left eye. A cherry red spot arises because the inner retina becomes edematous and opaque due to ischemia, whereas the fovea is composed predominantly of outer retinal layers, which are nourished by the choroidal circulation. As a result, the blood flow to the fovea is preserved, maintaining its coloration and creating a stark contrast with the surrounding ischemic retina. Optical coherence tomography (OCT) revealed swelling of the inner layer of the retina in the right eye, but there were no abnormalities in the left eye (Figure 2).

Fluorescein angiography showed no obvious delay in the retinal circulation time or inner retinal circulation time in the right eye, and no filling delay was observed in the cilioretinal artery, indicating preserved perfusion through this vessel. The Goldman perimetry test revealed a peripheral visual field defect in the right eye, accompanied by a residual central visual field. Clinically, the combination of acute painless unilateral vision loss, a cherry-red spot on fundus examination, and inner retinal swelling on OCT is highly suggestive of CRAO. The preservation of the central visual field and partial recovery of visual acuity, along with fundus findings showing a preserved retinal area from the optic disc to the macula, as well as the maintained circulation observed on fluorescein angiography, may suggest involvement of a cilioretinal artery. The blood test showed a high high-density lipoprotein (HDL) level of 126 mg/dL; however, there were no abnormalities detected in the other coagulation systems or autoimmune markers. The systemic examination, which included head magnetic resonance imaging + magnetic resonance angiography (MRI + MRA), echocardiography, and cervical vascular ultrasound, revealed hypoplasia of the right vertebral artery as the only abnormality. In this case, the patient presented more than one week after symptom onset, beyond the therapeutic time window for CRAO. Therefore, no specific treatment for CRAO was administered. The patient was managed with supportive care, close observation, and comprehensive systemic evaluation, without pharmacologic or surgical intervention.

In the initial post-examination period, marked by the first two months, there was an improvement in retinal edema in the right eye, accompanied by a recovery in retinal color tone. However, the optic disc exhibited a pale appearance. Visual acuity in the right eye demonstrated an enhancement to 0.5, yet no substantial alterations were observed in visual field impairment as measured by Goldmann perimetry when compared to the initial examination. After three to six months, the retina in the right eye underwent atrophy and thinning, accompanied by a progression in the pallor of the optic disc. Despite significant retinal structural deterioration, visual acuity was maintained at around 0.6 to 0.7, underscoring the role of collateral circulation from the ciliary artery.

**Figure 3:**
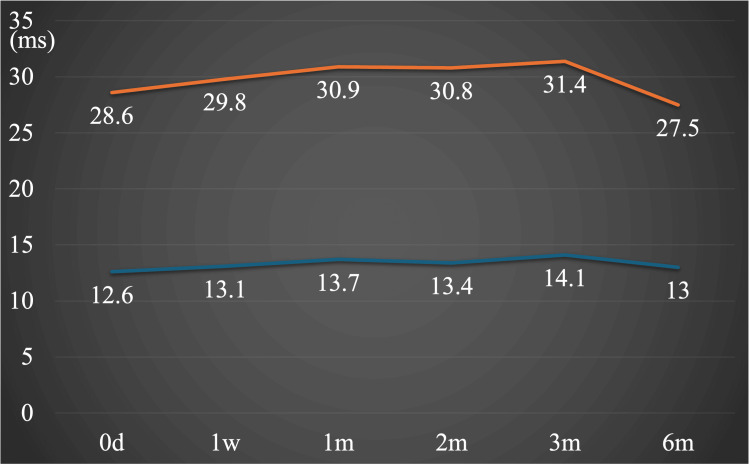
Light-adapted cone ERG a-wave and b-wave peak time over time. The a-wave and b-wave peak times showed no significant changes in the long term.

**Figure 4:**
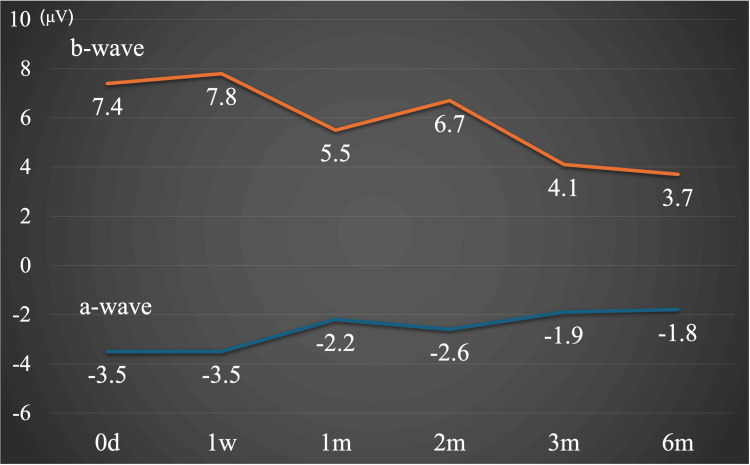
Light-adapted cone ERG a-wave and b-wave amplitudes over time. ERG: Electroretinography Both a-wave and b-wave amplitudes showed a long-term decreasing tendency.

In contrast to the fluctuations in visual acuity, the ERG's cone response parameters exhibited a tendency to decline over time, as evidenced by the amplitudes of the a-wave and b-wave (Figures 3, 4). 

These observations suggest that damage may have affected the inner layers of the retina and continued to progress after reperfusion. In this case, since the patient was a child, measurements were taken only under light-adapted conditions for simplicity, avoiding dark-adapted conditions. The photopic ERG was recorded according to the ISCEV standard [11]. The ERG proved to be a valuable tool for evaluating retinal function over time, providing a detailed understanding of the retina's overall condition. Table 1 summarizes the temporal changes in visual acuity, OCT measurements, and ERG parameters observed over the six-month follow-up period.

**Table 1:** Time course of visual acuity and OCT, ERG OCT: Optical coherence tomography, ERG: Electroretinography The inner retinal thickness was assessed by measuring from the internal limiting membrane (ILM) to the inner nuclear layer (INL). Manual measurements were performed perpendicular to the retinal pigment epithelium (RPE) at a location 1000 µm temporal to the foveal center.

		0 day	1 week	1 month	2 months	3 months	6 months
logMAR Visual Acuity		0.22	0.10	0.30	0.30	0.30	0.22
OCT	Total Retinal Thickness, μm	298	249	181	124	114	111
	Inner Retinal Thickness, μm	169	100	64	57	57	45
ERG	a-wave amplitue, μV	-3.5	-3.5	-2.2	-2.6	-1.9	-1.8
	b-wave amplitue, μV	7.4	7.8	5.5	6.7	4.1	3.7
	a-wave peak time, ms	12.6	13.1	13.7	13.4	14.1	13
	b-wave peak time, ms	28.6	29.8	30.9	30.8	31.4	27.5

This table provides a comprehensive overview of the patient's functional and structural retinal status at each assessment point. The patient adapted to the background light prior to testing. Sensor strips of skin electrodes were carefully placed 2 mm below the lower eyelid margin and connected to a lead wire. The stimuli consisted of flashing lights (intensity: 1.0 cd·s/m²) presented on a stable background illumination of 10 cd/m².

## Discussion

This paper presented a rare case of CRAO in a child, in which alterations in retinal function were evaluated over time using ERG. In the current case, diminished amplitude of the a-wave and b-wave under light-adapted conditions, which predominantly reflect inner retinal layer function [11]. Consequently, this observation indicates that central retinal artery occlusion may result in oxygen and nutrient deprivation in the inner layer, leading to bipolar cell dysfunction, a characteristic finding in CRAO. Kim et al. state that the a-wave impairment begins from light-adapted 3.0 (cones and OFF bipolar cells) and then progresses to dark-adapted 3.0 (combined) and dark-adapted 0.01 ERG (rods) in extreme CRAO, suggesting that in very severe cases, not only the inner retina but also the outer retinal layer could be damaged [4]. In adult CRAO cases, a-wave and b-wave amplitudes under light-adapted 3.0 conditions were preserved at approximately 60 to 90% of those in the unaffected eye [4]. In contrast, retinal function in our case was markedly reduced compared to age-matched normative data. To date, there have been no reports detailing the longitudinal pattern of ERG changes in CRAO. Although this case involves a pediatric patient and represents a single instance with presumed preservation of the cilioretinal artery, the findings suggest that developmental context should be carefully considered when interpreting ERG results in pediatric CRAO.

In this case, retinal atrophy and thinning, as well as the blanching of the optic disc, progressed over time, yet visual acuity remained relatively good. This phenomenon is hypothesized to be partly due to the intact status of the ciliary artery, which possesses a distinct blood supply route from the central retinal artery [12]. Consequently, the ciliary artery may contribute to the maintenance of retinal function in specific regions by circumventing circulatory disturbance. The stability of visual acuity in this case suggests that the presence of the ciliary retinal artery is clinically significant.

Furthermore, the evaluation of retinal function over time using the cone ERG was found to be useful for understanding the overall state of the retina, which cannot be determined from changes in visual acuity alone. Visual acuity only represents a part of the function of the eye and does not fully reflect the overall health of the retina [13]. Conversely, the ERG has been demonstrated to facilitate comprehensive examination of the retina, thereby serving as a pivotal instrument in understanding the progression of the disease and the efficacy of therapeutic interventions [14,15]. In this case, although visual acuity was relatively preserved, a persistent decline in ERG amplitudes was observed, indicating progressive retinal dysfunction and atrophy. These ERG findings suggest ongoing deterioration of retinal function and may serve as an early indicator of disease progression. Only cone ERG under light-adapted conditions was performed due to the patient’s age, and thus, rod system function could not be assessed. Nevertheless, the light-adapted ERG alone provided valuable insight into functional deterioration over time. Furthermore, although partial recovery of visual acuity was observed, the peripheral visual field defect documented by Goldmann perimetry remained unchanged throughout the follow-up period. This discrepancy highlights the limitation of visual acuity as a sole measure of retinal function, particularly in evaluating peripheral retinal damage. The parallel between sustained visual field loss and declining ERG amplitudes suggests a possible association between persistent clinical visual field deficits and widespread retinal dysfunction. These findings underscore the value of incorporating both ERG and visual field testing to gain a more comprehensive understanding of functional outcomes in pediatric CRAO. This underscores the potential of ERG as a diagnostic tool in rare diseases such as CRAO in children, facilitating a more comprehensive understanding of the condition and informing future treatment strategies. However, it is crucial to emphasize the necessity for further accumulation of cases and the conduct of more detailed research to deepen our understanding of these conditions and their prospects.

This study has several limitations. First, it is a single-case report, which limits the generalizability of the findings. Second, dark-adapted ERG responses were not obtained due to the patient’s young age, precluding evaluation of rod system function.

## Conclusions

In this case, we employed ERG to assess retinal function over time in a rare instance of CRAO that manifested in a pediatric patient. ERG analysis revealed functional alterations in the entire retina, which were not discernible through visual acuity alone. Notably, despite partial recovery of visual acuity, the peripheral visual field defect observed on Goldmann perimetry remained unchanged throughout the follow-up period. This finding, together with the progressive decline in ERG amplitudes, suggests a sustained association between functional retinal impairment and persistent clinical visual field deficits. These results further underscore the utility of ERG as a comprehensive tool for evaluating overall retinal function beyond central visual acuity alone. This finding underscores the utility of ERG as a valuable tool for deepening our comprehension of the pathophysiology of pediatric CRAO. Furthermore, the results suggest that the ciliary retinal artery may play a significant role in maintaining visual acuity. These findings are expected to contribute to the diagnosis and treatment of pediatric CRAO, and further accumulation of cases is anticipated to elucidate.
